# Bay breeze climatology at two sites along the Chesapeake bay from 1986–2010: Implications for surface ozone

**DOI:** 10.1007/s10874-013-9260-y

**Published:** 2013-06-30

**Authors:** Ryan M. Stauffer, Anne M. Thompson

**Affiliations:** Department of Meteorology, The Pennsylvania State University, University Park, PA 16802 USA

**Keywords:** Ozone, Bay Breeze, Mid-Atlantic, Climatology, Chesapeake Bay, Hampton, Baltimore, Nitrogen Oxides

## Abstract

Hourly surface meteorological measurements were coupled with surface ozone (O_3_) mixing ratio measurements at Hampton, Virginia and Baltimore, Maryland, two sites along the Chesapeake Bay in the Mid-Atlantic United States, to examine the behavior of surface O_3_ during bay breeze events and quantify the impact of the bay breeze on local O_3_ pollution. Analyses were performed for the months of May through September for the years 1986 to 2010. The years were split into three groups to account for increasingly stringent environmental regulations that reduced regional emissions of nitrogen oxides (NO_x_): 1986–1994, 1995–2002, and 2003–2010. Each day in the 25-year record was marked either as a bay breeze day, a non-bay breeze day, or a rainy/cloudy day based on the meteorological data. Mean eight hour (8-h) averaged surface O_3_ values during bay breeze events were 3 to 5 parts per billion by volume (ppbv) higher at Hampton and Baltimore than on non-bay breeze days in all year periods. Anomalies from mean surface O_3_ were highest in the afternoon at both sites during bay breeze days in the 2003–2010 study period. In conjunction with an overall lowering of baseline O_3_ after the 1995-2002 period, the percentage of total exceedances of the Environmental Protection Agency (EPA) 75 ppbv 8-h O_3_ standard that occurred on bay breeze days increased at Hampton for 2003–2010, while remaining steady at Baltimore. These results suggest that bay breeze circulations are becoming more important to causing exceedance events at particular sites in the region, and support the hypothesis of Martins et al. (2012) that highly localized meteorology increasingly drives air quality events at Hampton.

## Introduction

### Surface ozone (O_3_)

Surface O_3_ is a United States Environmental Protection Agency (EPA) regulated pollutant that has been shown to have adverse effects on the human respiratory system (e.g. Burnett et al. 1994; Jerrett et al. 2009) and photosynthesis in vegetation, leading to crop destruction (Krupa and Manning 1988; Fishman et al. 2010 and references therein). The O_3_ molecule is a secondary pollutant formed through a combination of nitrogen oxides (NO_x_), volatile organic compounds (VOCs), and sunlight, and its concentration near the surface has been shown to be dependent on several, often complex processes such as incoming solar radiation and cloud cover, temperature, precursor compound concentrations, wind speed, and boundary layer height (Comrie 1990; Sillman and Samson 1995; Bloomer et al. 2009; Steiner et al. 2010; Banta et al. 2011).

### The bay breeze

A bay or sea breeze (from here on, bay breeze) is a small-scale circulation that arises from a pressure gradient formed from the temperature contrast of air over land and air over water (Miller et al. 2003). Water has a specific heat capacity greater than land, thus much more energy is required to raise the temperature of a water body than a land surface. Since air is heated from below, temperature differences between the water surface and land surface result in a similar temperature gradient in the near-surface air. A low-level pressure gradient forms with higher air pressure over the water. Given that larger-scale background winds lack the forcing to oppose this local pressure gradient, air is forced from the water surface over the land (Simpson 1994). At night, when the land cools quicker than the water, a reversal of the pressure gradient and flow in the opposite direction initiates the land breeze.

### Motivation for study

The meteorological conditions needed to form a bay breeze and produce O_3_ go hand in hand. The combination of warm weather and intense sunlight needed to cause a temperature gradient from land to water can eventually lead to a bay breeze at coastal locations. The heat and incoming solar radiation, in the presence of high mixing ratios of NO_x_ and VOCs, can produce high amounts of O_3_ over land. The cooler water and adjacent air temperatures over water also result in lower boundary layer heights relative to farther inland areas (Berman et al. 1999), concentrating O_3_ in a smaller volume (Banta et al. 2005; Banta et al. 2011). The stagnant conditions necessary to allow a bay breeze to become the dominant circulation during the daytime also allows a buildup of O_3_ in the boundary layer due to lack of venting and the accumulation of pollutants (Rappenglück et al. 2008; Wu et al. 2010; Banta et al. 2011; Loughner et al. 2011). The bay breeze front can then transport O_3_-rich air masses and pollution well inland (Darby 2005; Lin et al. 2007). Previous studies have found the bay breeze to be a mechanism through which emissions and O_3_ from urban areas can be transported to more rural locations (Angevine et al., 2004; Darby et al. 2007; White et al. 2007).

In addition to similar meteorology controlling both O_3_ production and bay breeze initiation, the behavior of O_3_ over water surfaces is quite different than over land. While air masses originating from marine environments generally contain low O_3_, the land/bay breeze system transports morning terrestrial emissions and O_3_ over the water surface that then recirculate back to coastal locations (Banta et al. 2005). Ozone readily deposits to surfaces and vegetation over land, but its deposition velocity over water (~0.07 cm s^−1^) is five to six times slower than over a terrestrial (~0.4 cm s^−1^) surface (Lenschow et al. 1981; Lenschow et al. 1982; Hauglustaine et al. 1994; Wesely and Hicks 2000). This weaker deposition velocity produces less of a flux of O_3_ onto the water surface where it is effectively removed from the system. Because of this, high near-surface O_3_ mixing ratios may accumulate over the water in the morning when the land/bay breeze circulation is dominant. In addition to the reduced deposition of O_3_, minimal nighttime titration of O_3_ from a lack of NO over the water surface will decrease O_3_ loss, leading frequently to higher O_3_ observations over a water body at night (Mao et al. 2006).

Participation in two recent projects examined these effects and provided the motivation for a long-term historical analysis of bay breezes and O_3_. During the Chemistry of the Atmospheric Boundary Layer Experiment (CAPABLE; http://capable.larc.nasa.gov/) project in July 2010 in Hampton, Virginia (37.07º, −76.36º) near the mouth of the Chesapeake Bay, several instances of bay breezes were observed (Martins et al. 2012), with some of them leading to elevated O_3_ and violations of the EPA 8-h O_3_ mixing ratio standard of 75 ppbv. The 8-h O_3_ standard determines compliance with the National Ambient Air Quality Standard (NAAQS; Environmental Protection Agency, Ozone Air Quality Standards) and is therefore an important metric to evaluate during bay breeze events. The only two violations of the NAAQS at Hampton in July 2010 occurred on bay breeze days (Martins et al. 2012), when O_3_ spiked after the passage of the bay breeze front.

The Deriving Information on Surface Conditions from Column and Vertically Resolved Observations Relevant to Air Quality (DISCOVER-AQ; http://nasa.gov/discover-aq) project is a multiyear campaign to capture surface variability of air quality measurements with total column observations for satellite applications. The summer 2011 deployment in the Baltimore-Washington metropolitan area included several ground sites susceptible to bay breeze meteorology, with a number of NAAQS violations occurring during bay breeze events at Edgewood, Maryland (Stauffer et al. 2012). These observations impelled a hypothesis that as photochemical O_3_ precursor emissions decrease through regulation, local meteorology may play a defining role in coastal Mid-Atlantic air pollution events as has been shown on a case study basis in various locations (Banta et al. 2005, Martins et al. 2012, Stauffer et al. 2012).

A climatological analysis of surface O_3_ on bay breeze days is performed using observations from 1986–2010, and accounts for changes in NO_x_ which have strong effects on surface O_3_. We attempt to answer the question raised by Martins et al. (2012), namely whether exceedances on days that exhibit a bay breeze correspond to a growing portion of total NAAQS violations at particular locations. To our knowledge, this study represents the first time a combined climatological analysis of bay or sea breezes and surface O_3_ has been performed. The analysis is performed using meteorological and surface O_3_ data at Hampton, Virginia and Baltimore, Maryland, two sites near the Chesapeake Bay.

## Methodology

### Measurement sites

In order to perform this study, closely located meteorological and O_3_ measurements with sufficient historical records are needed. Several coastal Chesapeake Bay locations were identified where hourly surface meteorological measurements (National Climatic Data Center, National Oceanic and Atmospheric Administration, U.S. Department of Commerce) and data from a nearby O_3_ monitoring station were available. Whereas studies of Chesapeake Bay breeze climatology have been performed before (e.g. Sikora et al. 2010), the results have never been combined with surface O_3_ data.

**Fig. 1:**
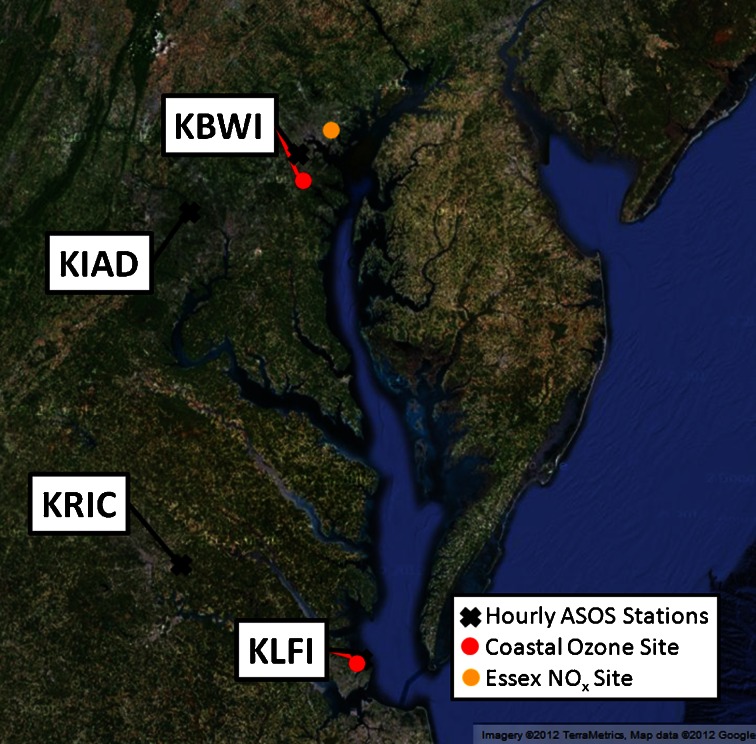
Chesapeake Bay region and study locations showing ASOS stations (*black crosses*) used for meteorological analysis, surface O_3_ monitors (*red dots*) and the Essex NO_x_ monitor (*orange dot*)

The airport at Aberdeen Proving Ground (KAPG) and Baltimore-Martin State Airport (KMTN) were both considered for analysis, but the spotty reporting of hourly meteorological variables made the data insufficient for characterizing bay breeze events. Longstanding records of both O_3_ and meteorological variables were found at Hampton, Virginia (KLFI) and Baltimore, Maryland (KBWI) and these were chosen for bay breeze analysis. Locations of all sites used in this study are presented in Fig. 1.

Baltimore is the largest city in Maryland with a population of over 600,000 residents, and is located within and affected by emissions from the Baltimore-Washington Metropolitan Area, a region with nearly 9 million people. Hampton, Virginia, is a moderately urbanized area with population near 150,000, located within the Hampton Roads region of southeastern Virginia with ~1 million residents. The differences in total population both in the cities themselves and regionally affect the total anthropogenic NO_x_ emissions, which aid in O_3_ production. Baltimore emitted a total of ~17,000 metric tons of nitrogen oxides compared to ~3,200 metric tons at Hampton for the year 2008 (Environmental Protection Agency 2008, National Emissions Inventory). These differences were considered when evaluating and comparing surface O_3_ mixing ratios at each location.

### Bay breeze criteria

**Fig. 2:**
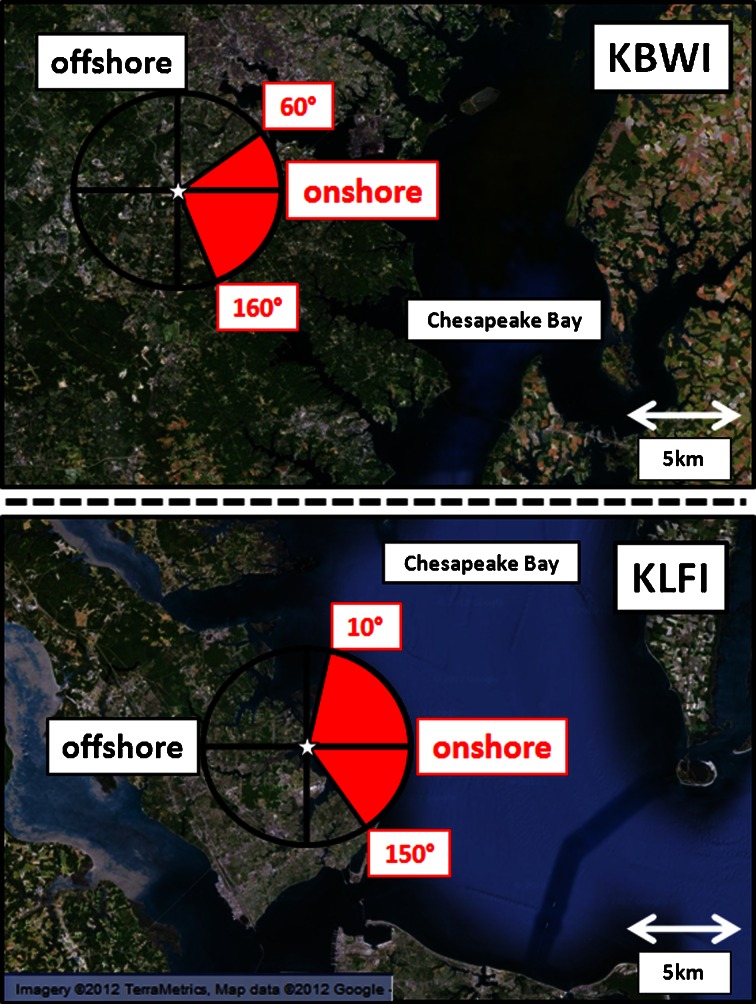
A close-up map of coastal sites with bay breeze wind directions defined as onshore (*red shading*) and offshore (*no shading*). Hourly wind directions are reported to the nearest ten degrees

Hourly surface meteorological measurements were analyzed from KLFI and KBWI to determine bay breeze events. A higher resolution map of each site, along with what are defined as onshore and offshore wind directions, is shown (Fig. 2). Following Sikora et al. (2010), a station located inland, enough so as to be unaffected by the bay breeze, was picked for both sites as an additional reference in determining bay breeze days. These sites are Richmond International Airport (KRIC; for Hampton, VA) and International Airport at Dulles (KIAD; for Baltimore, MD). Table 1 shows the meteorological Automated Surface Observing Systems (ASOS) and their respective International Civil Aviation Organization (ICAO) airport codes for the sites used in this study.

**Table 1:** Airport ASOS sites used in this study for determination of bay breeze events and day types

Hourly ASOS Station	ICAO Code	Latitude (º)	Longitude (º)	Elevation (m)	Dist. Inland (km)
Balt.-Wash. Intl. Airport	KBWI	39.18	−76.67	44.5	13
Dulles Intl. Airport	KIAD	38.95	−77.46	95.0	85
Langley Air Force Base	KLFI	37.08	−76.36	3.4	6
Richmond Intl. Airport	KRIC	37.51	−77.32	50.9	94

Hourly surface O_3_ data (Environmental Protection Agency, Remote Sensing Information Gateway (RSIG) from the monitor locations were used to perform a climatological analysis from 1986–2010. The O_3_ sites used for this analysis along with their Federal Information Processing Standard (FIPS) codes, which identifies the specific instrument site, are shown in Table 2. The Hampton, VA, O_3_ monitor was moved 10 km NE in 2009 and 1 km N again in 2010 for the CAPABLE field project, but these movements are not considered to have had a noticeable effect on the measured O_3_ values since measurements at closely located O_3_ monitors at many locations susceptible to bay breeze circulations are very well correlated (Maryland Department of the Environment, Maryland 5-year network assessment). Study periods from each year were limited to May-September for this paper, when the sun provides sufficient radiation to put Mid-Atlantic U.S. sites at greatest risk for exceedances from photochemical production of O_3_.

**Table 2:** Ozone monitoring sites used in this study for analysis of surface O_3_ by day type

Ozone Monitoring Site	FIPS Code	Latitude (º)	Longitude (º)	Dates Active
Baltimore, MD	240030014	38.9025	−76.6531	1981–Present
Hampton, VA	516500004	37.0033	−76.3992	1981–2009
Hampton, VA	517000013	37.0998	−76.4811	2009
Hampton, VA	516500008	37.1037	−76.3870	2010–Present

Days were separated into three day types: 1) Bay Breeze Days, 2) Non-Bay Breeze Days and 3) Rainy/Cloudy Days. Rainy or cloudy days were separated from all other days to keep from introducing a low bias in O_3_ on days when a bay breeze did not occur. Cloud cover greatly reduces the photochemical production of O_3_, suppressing mixing ratios. Additionally, rainfall will quickly wash out O_3_ and O_3_ precursors through wet deposition. The goal of separating days into these three types was to minimize the discernible meteorological differences between non-bay breeze and bay breeze days.

**Fig. 3:**
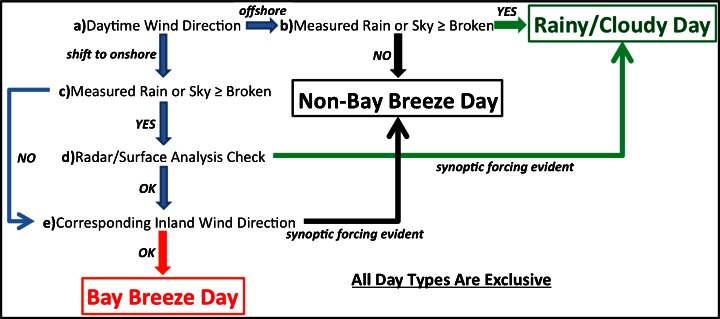
Bay Breeze criteria flow chart. Criteria checks begin at **a**) and continue as shown until a day type is determined. Full explanations for each criterion are described in the text

The method for picking each day type at both sites is outlined in Fig. 3. For each day, the daytime (0900 to 1600 Eastern Standard Time, EST) wind directions were evaluated (Fig. 3a). If the hourly wind direction measurement changed from either offshore (160º to 360º at KLFI; 170º to 50º at KBWI), calm, or light and variable, to onshore (10º to 150º at KLFI; 60º to 160º at KBWI) sustained for two or more consecutive hours during the period, the next step was evaluated. If this wind shift to onshore directions did not occur (Fig. 3b), then the day was either marked non-bay breeze or rainy/cloudy based on rainfall and cloud cover measurements. The latter category meant average daytime sky coverage was greater than “broken” with 7/8 or more cloud fraction, or there was measurable rainfall during the daytime. If the winds shifted to onshore during the day, the daytime cloud cover and rainfall were evaluated (Fig. 3c). If skies were less than broken and there was no measureable rainfall during the day, the final check was performed (Fig. 3e). If an average of broken skies or rainfall was recorded in conjunction with the bay breeze direction wind shift, radar and surface charts were manually analyzed (Fig. 3d; University Corporation for Atmospheric Research, Image archive meteorological case study selection kit; Plymouth State Weather Center, Plotted Surface Data Maps). Days that exhibit bay breezes can often breed localized thunderstorm activity, so a closer inspection is warranted when rainfall is measured. If there was no evidence of a large-scale circulation causing the wind shift to onshore directions, then the final check for a bay breeze day could be evaluated. Otherwise the day was placed in the rainy/cloudy day type. For the final criterion, the corresponding wind direction and speed were checked at the respective inland surface station (Fig. 3e; KRIC for Hampton; KIAD for Baltimore). This check was performed to attempt to eliminate synoptic-scale winds that were observed by both stations, indicating a larger-scale effect. If the corresponding inland wind directions were not from the same onshore wind directions for two or more hours or wind speeds were less than 3 ms^−1^ from any direction, then the day was grouped with bay breeze days. If the winds were from the same onshore directions at 3 ms^−1^ or more, the day was placed with non-bay breeze days. The 3 ms^−1^ speed was chosen to offset the chance that inland wind directions were random or light and variable during the day but still recorded hours of onshore wind directions; a less likely coincidence at higher wind speeds.

All available days from May through September, for the years 1986–2010 were placed into one of the three day types illustrated in Fig. 3. These day types were used to separate and analyze the behavior of surface O_3_ for each group of days.

### NO_x_ emission reductions

Regulations of NO_x_ emissions from power plants, a precursor for O_3_ production, were implemented in the early 2000s. Levels of NO_x_ across the United States dropped and O_3_ levels responded in kind (Kim et al. 2006). Frost et al. (2006) also found that by 2003, NO_x_ emissions from 53 eastern U.S. power plants had been reduced by 50 % from 1999 levels. For these reasons, 2002 has been used as a cut off between previous years and the current lower NO_x_ regime when analyzing historical O_3_ records (i.e. Bloomer et al. 2009).

**Fig. 4:**
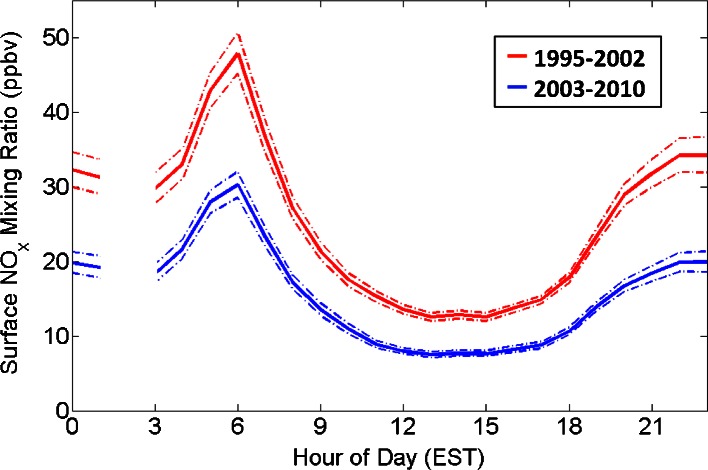
Average diurnal surface mixing ratios of NO_x_ at Essex, MD broken into May-September, 1995–2002 and 2003–2010. *Dashed lines* represent bounds on the 95 % confidence limits. Data at 02 EST are absent due to nightly instrument span checks greatly reducing the number of available measurements

The result of NO_x_ emissions reductions is noticed at the Essex, MD surface NO_x_ monitor, used here as a representative for the region, located on the north shore of the Chesapeake Bay ~40 km from the Baltimore O_3_ monitor (Fig. 4). Daily averaged NO_x_ mixing ratios were reduced by nearly 40 % from the years 1995–2002 to 2003–2010, May-September. Surface NO_x_ mixing ratios were also statistically different beyond 95 % confidence for every hour of the day between the two periods. Statistical significance was determined from a statistical bootstrap resampling method performed 10,000 times (Efron 1979; Efron and Tibshirani 1993).

Based on Bloomer et al. (2009)’s use of the year 2002 to distinguish transitioning Mid-Atlantic NO_x_ regimes, the O_3_ datasets are split into three roughly equal length periods: 1986–1994, 1995–2002, and 2003–2010. While the NO_x_ emissions from year to year within each period are not constant, this method of splitting the data ensures that O_3_ measurements within each period are at least comparable and can be analyzed together.

**Fig. 5:**
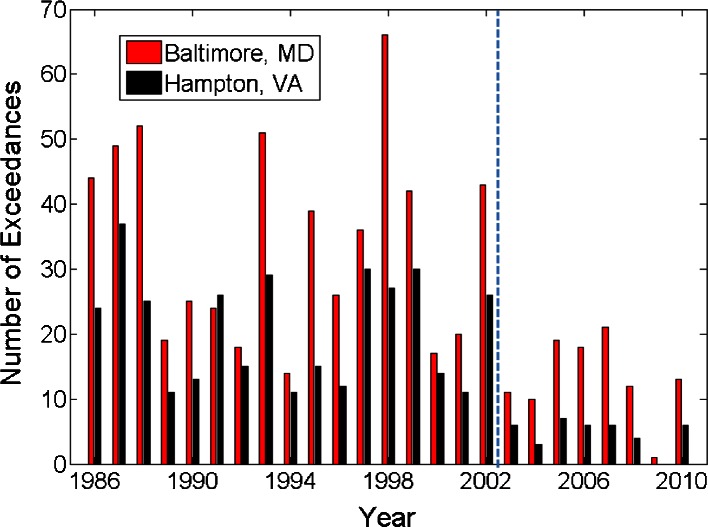
Total number of exceedances of the 8-h NAAQS standard of 75 ppbv from May- September for each year from 1986–2010. A vertical dashed line separates 2002 and 2003, the delimiting year for the most recent study periods

The effects of the transition in NO_x_ regimes on surface O_3_ can be seen in the total number of exceedances at both sites by year in Fig. 5. For uniformity, though the standard has been decreased in recent years, exceedance in this paper is defined by the current NAAQS definition of greater than 75 ppbv O_3_ for an 8-h average. The number of exceedances in the most recent study period at Baltimore and Hampton decreased dramatically from 2002 and prior.

## Results

### Bay breeze days at each site

**Fig. 6:**
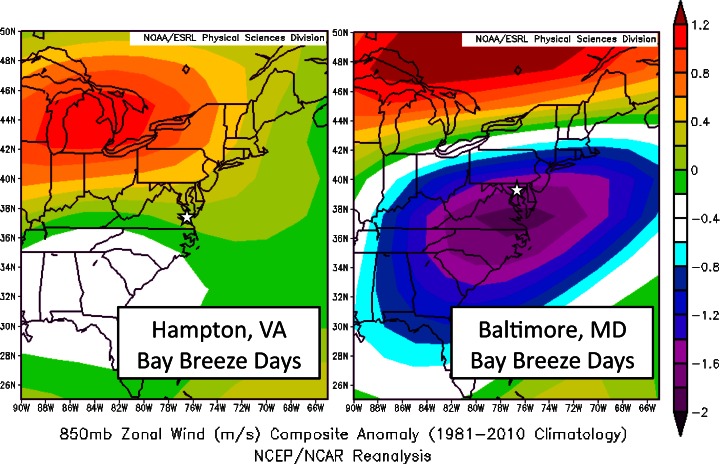
850 hPa zonal wind anomalies compared to the 1981–2010 climatology for all bay breeze days at each site. Stars mark each location

Fewer bay breeze days were recorded at Baltimore (343 days; KBWI is 13 km inland) than at Hampton (643 days; KLFI is 6 km inland) for 1986–2010. Farther inland penetration and propagation of the bay breeze front requires weaker opposing forces. Chiba et al. (1999) describe 850 hPa cross-shore winds as a dominant factor determining inland penetration of the water-body breeze. Figure 6 shows the 850 hPa zonal (the Chesapeake Bay coastline is approximately North–south oriented) wind anomalies for each site’s bay breeze days“(Earth System Research Laboratory (ESRL) Physical Sciences Division, Daily Mean Composites). Much weaker opposing 850 hPa winds are necessary for the bay breeze to reach KBWI than at KLFI due to the combination of the site’s proximity to the coast, as well the typically cooler waters near the mouth of the bay, making these exceptional conditions less of a requirement; thus, 850 hPa zonal winds are about average on KLFI bay breeze days. Additionally, there are fewer wind directions considered “onshore” at KBWI than at KLFI, likely leading to fewer bay breeze events.

### Day type meteorology

A goal of the bay breeze day identification process was to reduce as much as possible the meteorological differences between bay breeze and non-bay breeze days. Following analyses by Camalier et al. (2007), who determined that maximum daily temperature and average midday relative humidity were the two dominant meteorological variables connected to surface O_3_ variability and trends in the Mid-Atlantic U.S., a statistical analysis was performed on the hourly meteorological measurements at both sites to evaluate differences between bay breeze days and non-bay breeze days. Along with daily maximum temperature and midday (1000–1600 EST) relative humidity, average daytime (0600–1800 EST) cloud cover was added to assess possible differences in incoming solar radiation for each day type. The only variable that showed a statistically significant difference between the day types was the average midday relative humidity at KBWI (Table 3). Camalier et al. (2007) estimated less than a 1 % decrease in surface O_3_ per 1 % increase in relative humidity, whereas surface O_3_ was found to increase by approximately 4 % per 1 °C increase in maximum temperature. With a small statistical difference (0.4 %) in relative humidity between bay breeze days and non-bay breeze days at Baltimore, the impact on surface O_3_ mixing ratios amongst the day types is expected to be minimal when considering all the meteorological measurements.

**Table 3:** Pertinent meteorological variables with 95 % confidence intervals around the mean. Values are separated by day type and checked for statistically significant differences beyond 95 % confidence. Cloud cover is converted from reported octas to percentages

KLFI (Hampton, VA)	Max Temperature (°C)	Cloud Cover (%)	Daytime RH (%)
Bay Breeze	28.4–29.0	42.5–46.4	55.4–57.2
Non-Bay Breeze	28.2–28.5	45.8–47.9	55.5–56.6
Statistically Significant?	NO	NO	NO
KBWI (Baltimore, MD)	Max Temperature (°C)	Cloud Cover (%)	Daytime RH (%)
Bay Breeze	28.5–29.3	47.8–52.6	48.3–50.4
Non-Bay Breeze	28.5–28.9	48.9–51.0	46.9–47.9
Statistically Significant?	NO	NO	YES

### O_3_ by day type

**Fig. 7:**
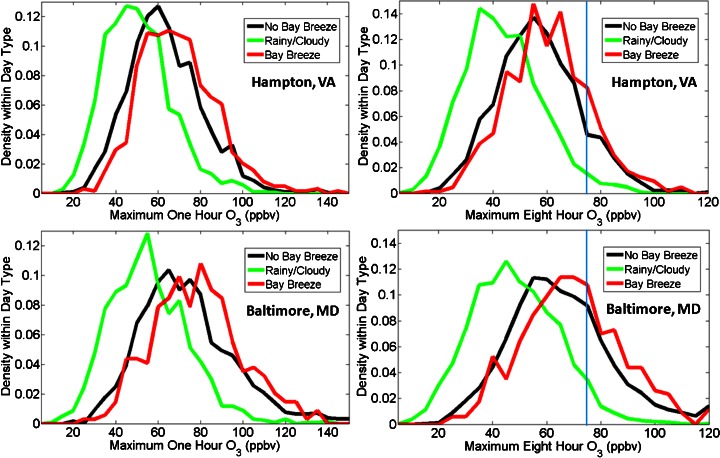
Histograms of maximum 1- (*left column*) and 8-h (*right column*) surface O_3_ averages in density by day type for May-September, 1986–2010 for non-bay breeze (*black*), rainy/cloudy (*green*), and bay breeze (*red*) days. The day types were separated and densities calculated within each group. *Vertical blue* lines on 8-h O_3_ figures mark the 75 ppbv NAAQS standard. Data are binned every 5 ppbv

The 1-h O_3_ maxima for each day type at both sites were calculated and exhibit a skewed distribution (Fig. 7) that is typically observed with surface O_3_. Prior to calculating the maximum 1-h average for each, 75 % error-free hourly averages for the whole day (18 of 24 measurements) were required to ensure a true representative maximum is reported for that day. A small number of extremely high averages are seen in exceptional cases, and for the entire 25 year study period the mean maximum 1-h average was higher on bay breeze days than non-bay breeze days at both locations (69.1 ± 18.2 vs. 63.4 ± 17.5 ppbv at Hampton; 78.0 ± 21.2 vs. 73.3 ± 22.3 ppbv at Baltimore, respectively). A larger spread in values is noticeable in the analysis of Baltimore, MD 1-h O_3_ maxima, as that location sees many more elevated O_3_ days than Hampton, VA, the latter evidenced by a more narrow distribution.

The density distribution of all maximum 8-h averages by day type for each site is also shown in Fig. 7. In addition to the requirements for calculating a 1-h O_3_ average, 8 of 12 valid hourly averages from 09–20 EST were required prior to calculating an 8-h O_3_ maximum. This requirement is to keep from reporting 8-h maxima on days with only nighttime data, which are likely too low and not representative of the actual O_3_ mixing ratios on that day.

The distributions of the maximum 8-h averages for each day type at both locations show similar behavior to the maximum 1-h averages. Again, the bay breeze days exhibit the highest mean maximum 8-h O_3_ averages, followed by the non-bay breeze days and the rainy/cloudy days. Bay breeze days at Hampton had a mean 8-h maximum of 60.4 ± 15.3 versus 56.9 ± 15.4 ppbv on non-bay breeze days, while bay breeze days at Baltimore had a mean 8-h maximum of 69.2 ± 18.4 versus 65.1 ± 18.9 ppbv on non-bay breeze days. The higher 8-h averages on bay breeze days have regulation implications because the NAAQS standard of 75 ppbv is based on an 8-h running average.

A breakdown of O_3_ averages and day types are provided in Table 4. The data are separated into the three study periods of 1986–1994, 1995–2002, and 2003–2010 with number of each day type, number of exceedances per year and mean O_3_ averages at Hampton and Baltimore (note that the first study period contains one additional year compared to the two most recent periods). The sharp decrease in number of exceedances, as well as the average O_3_ values after 2002 can also be seen in Table 4. The reduction in NO_x_ emissions has reduced the frequency of regional pollution episodes, potentially increasing the pertinence of small-scale bay breeze events to air quality violations through recirculation of local emissions.

**Table 4:** Breakdown of surface O_3_ maximum averages and standard deviations, exceedances, and day types for both sites by study period from May to September, 1986–2010. Exceedance percentage is defined by (exceedances in day type)/(total occurrences of day type)

Hampton, VA	# of Days	Exceedances/year	Exceedance %	Avg. 1 h max (ppbv)	Avg. 8 h max (ppbv)
1986–1994					
Bay Breeze	215	5.3	22.3	73.2 ± 18.8	62.6 ± 15.7
No Bay Breeze	756	13.4	16.0	66.6 ± 18.5	59.2 ± 16.2
Rainy/Cloudy	375	2.1	5.1	53.9 ± 17.0	46.2 ± 15.2
1995–2002					
Bay Breeze	184	5.1	22.3	71.8 ± 18.1	63.3 ± 16.3
No Bay Breeze	646	14.0	17.3	66.4 ± 18.1	59.8 ± 15.9
Rainy/Cloudy	362	1.4	3.0	51.0 ± 15.6	44.2 ± 14.3
2003–2010					
Bay Breeze	244	2.0	6.6	63.4 ± 15.3	56.3 ± 13.3
No Bay Breeze	637	2.3	2.8	56.8 ± 13.4	51.2 ± 11.9
Rainy/Cloudy	310	0.4	1.0	46.0 ± 13.3	40.4 ± 12.6
All Years					
Bay Breeze	643	4.2	16.3	69.1 ± 17.9	60.4 ± 15.3
No Bay Breeze	2,039	10.0	12.3	63.4 ± 17.5	56.9 ± 15.4
Rainy/Cloudy	1,047	1.3	3.2	50.6 ± 15.8	43.8 ± 14.3
Baltimore, MD	# of Days	Exceedances/year	Exceedance %	Avg. 1 h max (ppbv)	Avg. 8 h max (ppbv)
1986–1994					
Bay Breeze	120	5.2	39.4	80.2 ± 21.4	70.2 ± 18.3
No Bay Breeze	721	25.1	31.4	76.8 ± 23.5	67.6 ± 19.7
Rainy/Cloudy	329	1.4	4.0	55.1 ± 18.4	46.6 ± 15.9
1995–2002					
Bay Breeze	132	7.0	42.4	81.5 ± 21.4	72.5 ± 19.8
No Bay Breeze	607	24.4	32.1	75.9 ± 22.8	67.3 ± 19.8
Rainy/Cloudy	459	4.0	7.0	57.9 ± 19.4	50.2 ± 17.1
2003–2010					
Bay Breeze	91	2.1	18.7	69.9 ± 16.1	63.1 ± 14.7
No Bay Breeze	524	8.0	12.2	65.8 ± 17.0	58.9 ± 14.9
Rainy/Cloudy	575	2.8	3.8	54.2 ± 17.1	47.7 ± 15.3
All Years					
Bay Breeze	343	4.8	35.0	78.0 ± 20.6	69.2 ± 18.4
No Bay Breeze	1,852	19.4	26.2	73.3 ± 22.1	65.1 ± 18.9
Rainy/Cloudy	1,363	2.7	4.9	55.6 ± 18.3	48.3 ± 16.1

### Baltimore and Hampton ozone diurnal differences

**Fig. 8:**
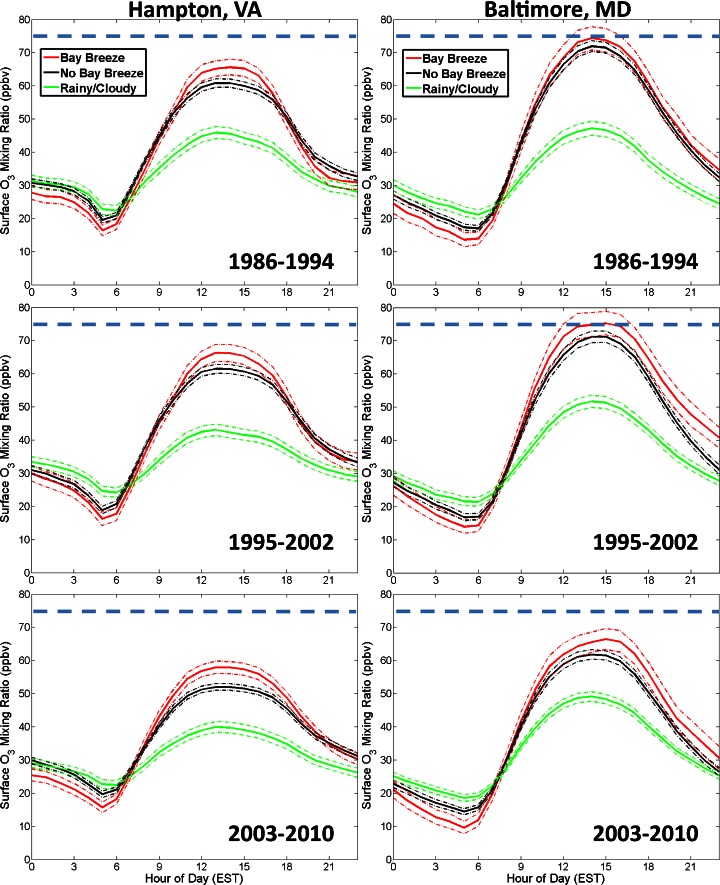
Average hourly surface O_3_ at Hampton (*left panels*) and Baltimore (*right panels*) by study period from May-September. Day types are separated into bay breeze (*red*), non-bay breeze (*black*), and rainy/cloudy (*green*) days. The dashed lines represent the 95 % confidence interval for each mean and the blue dashed line marks the 8-h NAAQS standard of 75 ppbv

To examine the behavior of O_3_ on each day type throughout the day, O_3_ values are placed into bins for each hour and averaged to obtain a diurnal cycle. The day types are separated and shown by study period at each site in Fig. 8. A statistical bootstrap method was performed 10,000 times to assess significance of the diurnal means for each hour. The 95 % confidence intervals at which statistical significance is determined are marked in Fig. 8, revealing differences among the bay breeze’s effect at the two sites.

At Hampton, on average, the bay breeze provides statistically significantly higher afternoon O_3_ than on non-bay breeze days for all three study periods. At Baltimore, the days with a bay breeze on average exhibit statistically significantly higher O_3_ only in the mid-afternoon hours of the 2003–2010 period (excluding the late evening hours of the 1995–2002 period). This result, along with the much greater differences in O_3_ mixing ratios amongst the day types in 2003–2010 at Hampton, supports the hypothesis that bay breeze circulations are causing higher anomalies from baseline O_3_ since the inception of the recent NO_x_ regulations. The diurnal cycles of O_3_ also show intriguing results related to the early morning meteorological conditions of each day type, with statistically significant differences in O_3_ during those hours as well. This will be discussed further below.

In addition to the greater day type differences in diurnal O_3_ noticed in 2003–2010, the typical time of day when non-bay breeze day and bay breeze day O_3_ diverge and become statistically different also changes in 2003–2010. The earliest statistical difference in O_3_ at Hampton occurs at 12 EST in both the 1986–1994 and 1995–2002 periods. In the 2003–2010 period, the hourly O_3_ is significantly different by 09 EST. The differences in timing when the non-bay breeze day and bay breeze day O_3_ diverge may indicate the effects that regional emissions reductions have on mid-morning O_3_. With reduced regional NO_x_, there are different behaviors between the regionally produced O_3_ on non-bay breeze days and the O_3_ produced under the extremely favorable conditions on bay breeze days. At Baltimore, the first statistically significant difference in daytime (again excluding the late evening hours of the 1995–2002 period) O_3_ occurs at 15 EST in the 2003–2010 period; much later than Hampton. This later O_3_ peak during bay breeze events has also been noted during DISCOVER-AQ at Edgewood, MD (Stauffer et al. 2012), and is likely a result of the recirculation of locally produced O_3_ and O_3_ precursors into the evening hours (Banta et al. 2005).

### Normalized ozone

In addition to the comparison of the average diurnal cycles of O_3_ for each day type, a direct comparison of normalized data is also performed. This eliminates the seasonality of surface O_3_ within each year and gives a measure of the anomalies from mean O_3_ displayed from the three day types throughout the O_3_ season.

To remove the seasonality of O_3_, the May through September season is broken down into eight sequential subsets. Seven groups contain 19 consecutive days each, with the last group containing the remaining 20 days. The eight sequential sets of days are formed to group dates with similar solar zenith angles together, damping the seasonal variations in surface O_3_. The entire O_3_ dataset falls into 24 separate groups at both sites when considering the three study periods. Within each of the 24 groups of O_3_ data, the average $\left(\overline{x}\right)$ O_3_ and standard deviation (***σ***) for each hour of the day is calculated, and the original data (*x*) in the group are normalized via the equation:

$$ A=\frac{x-\overline{x}}{\sigma } $$

Here, A is the surface O_3_ anomaly in standard deviation from the mean. Every O_3_ measurement is now in terms of anomaly from the mean for its respective hour of the day. This method of normalizing the data allows for a direct comparison of each day type with the mean O_3_ in that group since the original data are approximately normally distributed.

**Fig. 9:**
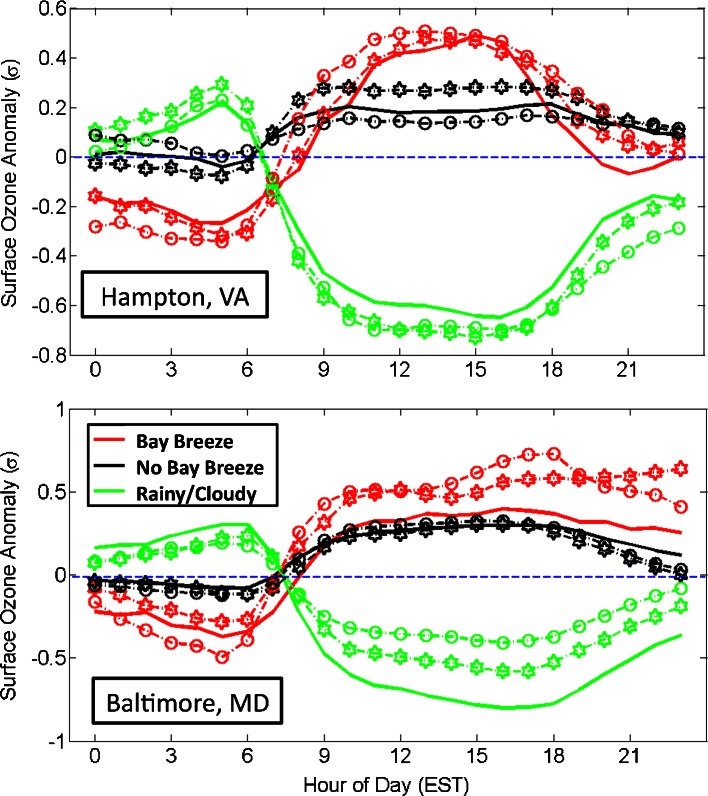
Average O_3_ anomaly in standard deviation from the mean by hour of day for each day type. Day types are separated into bay breeze (*red*), non-bay breeze (*black*), and rainy/cloudy (*green*) days. Study periods are also separated by 1986–1994 (*solid line*), 1995–2002 (*stars*), and 2003–2010 (*open circles*). The *zero line*, representing mean O_3_, is marked with a *dashed blue line*. Note the different y-axis for each plot

The O_3_ anomalies by hour of the day at both sites by day type show that rainy/cloudy days consistently have higher O_3_ in the morning hours compared with the mean (Fig. 9). This result is likely an outcome of a disturbed surface layer from higher early morning wind speeds on days with rain or cloud cover (01–06 EST wind speed average KLFI: 3.4 ms^1^, 95 % CI: 3.2–3.5 ms^−1^; KBWI: 2.1 ms^−1^, 95 % CI: 2.1–2.2 ms^−1^). Under these windier conditions, a stable nocturnal layer does not form, and early morning NO_x_ emissions do not titrate and suppress O_3_ mixing ratios to near zero. The opposite is true for bay breeze days. We find more stagnant (01–06 EST wind speed average KLFI: 2.2 ms^1^, 95 % CI: 2.1–2.3 ms^−1^; KBWI: 1.6 ms^−1^, 95 % CI: 1.4–1.7 ms^−1^), cloud-free conditions in place prior to bay breeze formation, leading to radiational cooling at night and a well-defined, undisturbed stable surface layer. In this well-defined stable layer, NO_x_ emissions are trapped and tend to titrate O_3_ relatively quickly until sunrise. Ozone also readily deposits to the surface in the shallow stable layer, leading to the lower morning O_3_ mixing ratios on bay breeze days. The non-bay breeze days fall in between those two results. Once the sun begins photochemically producing O_3_ on bay breeze and non-bay breeze days, the rainy/cloudy days fall below the mean because O_3_ production is inhibited.

Baltimore and Hampton show different qualities in the diurnal variability of O_3_ anomalies, especially late in the day. With respect to the mean, the high O_3_ on bay breeze days at Hampton subsides more quickly than at Baltimore, which remains elevated through the evening hours. At both sites however, the 2003–2010 bay breeze days represent the highest average anomalies during the daytime; further proof that localized meteorology plays a larger role in the current lower NO_x_ regime. At Baltimore, the 2003–2010 rainy/cloudy days represent less of a negative anomaly in O_3_ than in the earlier periods, likely because of a combination of overall lowering of O_3_ mixing ratios due to NO_x_ reductions, and a higher number of rainy/cloudy days analyzed in the 2003–2010 period. This same trend is not evident in the Hampton data, perhaps due to the smaller reduction in baseline O_3_ and fewer rainy/cloudy days at that site.

### Increasing role of the bay breeze at Hampton

To quantify the part that the bay breeze plays in the total number of exceedances at each site studied and how that changes with each study period, the total number of days with an 8-h average above 75 ppbv was calculated. The exceedances were then grouped by day type, and by study period. The number of bay breeze day exceedances was then compared with the total number of exceedances:

$$ \mathrm{Bay}\;\mathrm{Breeze}\;\mathrm{Exceedance}\%=\frac{\#\mathrm{Bay}\;\mathrm{Breeze}\;\mathrm{Exceedance}}{\#\mathrm{Total}\;\mathrm{Exceedance}\mathrm{s}} $$

**Fig. 10:**
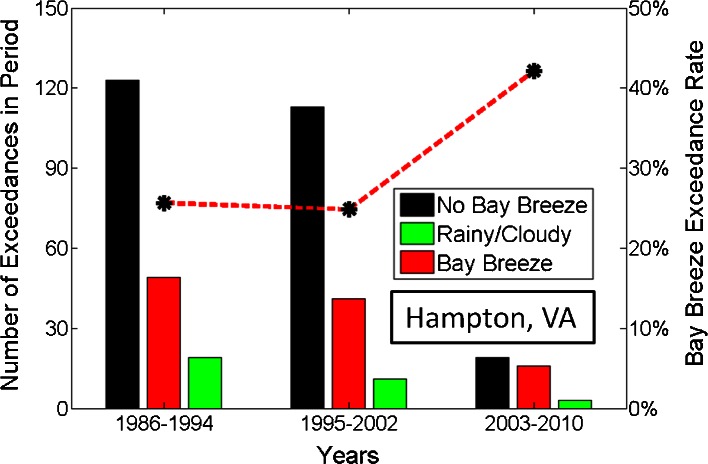
Bay breeze exceedance rate (defined as bay breeze exceedances divided by total exceedances) by study period. Total number of exceedances for each study period are shown broken down by non-bay breeze (*black*), bay breeze (*red*) and rainy/cloudy (*green*) days. The *red dashed line* with *black markers* represents the bay breeze exceedance rate for each study period, shown on the right y-axis

Figure 10 shows the results of distributing exceedances at Hampton by study period and evaluating the bay breeze exceedance percentage for each. At Hampton, the bay breeze exceedance rate holds at 25.5 % and 25.0 % for the first two periods then jumps to 43.2 % for 2003–2010. This result shows that the bay breeze has indeed become a larger factor in Hampton, VA, exceedances as suggested by Martins et al. (2012). At Baltimore, the bay breeze exceedance rate holds steady throughout the three study periods, 16.4 % (1986–1994), to 19.8 % (1995–2002), to 16.5 % (2003–2010) from past to present. At Baltimore, although the bay breeze leads to the highest O_3_ anomalies in the 2003–2010 time period, it does not significantly affect the percentage of exceedances.

### Discussion of exceedance rate differences

Since the reduction of NO_x_ emissions in the early 2000s, the bay breeze has contributed more to violating the EPA 8-h O_3_ standard at Hampton, VA than at Baltimore, MD. There are several possible reasons for this difference. Baltimore observed an average of almost 13 exceedances per year from May through September 2003–2010, while Hampton averaged fewer than 5 per year over the same period. Weather that is conducive to surface O_3_ violations appears to be more critical at Hampton than at Baltimore where baseline O_3_ and NO_x_ emissions are higher, and in the case of the former, bay breezes provide those exceptional conditions. This leads to the higher bay breeze percentage of total exceedances observed at the Hampton site. The recirculation of local O_3_ precursors at the Baltimore site may be overshadowed by the total regional emissions in the more urbanized Baltimore/Washington D.C. area. These reasons likely contributed to the lack of response in bay breeze exceedance percentage at Baltimore in the 2003–2010 period.

## Conclusions

In general, bay breezes in the Chesapeake Bay region are found to enhance air quality problems. Mean calculated 8-h O_3_ maxima were 3 to 5 ppbv higher on bay breeze days compared to non-bay breeze days at both sites in any given year period (Table 4). In both locations, afternoon mean hourly surface O_3_ was highest on bay breeze days compared to non-bay breeze and rainy/cloudy days for all time periods. The difference was statistically significant at Hampton, VA for afternoon hours during all three study periods, but was only statistically different during late afternoon hours for the 2003–2010 study period at Baltimore, MD (Fig. 8). Surface O_3_ anomalies were also calculated at each site by hour of day. Both locations observed the highest O_3_ anomalies during bay breeze days for the years 2003–2010 during the afternoon period (Fig. 9), showing the enhanced role of localized meteorology in the current lower NO_x_ regime.

The percentage of total exceedances during bay breeze days was steady at Baltimore for all three study periods, but jumped a total of 18.2 % from 1995–2002 to 2003–2010 at Hampton, with 43.2 % of exceedances occurring on bay breeze days in the latest period (Fig. 10). This result suggests an overall higher baseline O_3_ at Baltimore, where exceptional meteorological conditions are not as necessary for elevated O_3_ as they are at Hampton. This result also validates the hypothesis put forth by Martins et al. (2012) that the bay breeze is becoming more important to exceedance probability at certain locations, and will likely continue to be should O_3_ standards become more stringent.

It would be interesting to perform this type of analysis in other locations with air quality issues that are susceptible to water-body breezes. Other candidates for this type of analysis include Wallops Island, VA, which has ozonesonde records dating to 1970 and would be useful for examining the vertical structure of O_3_ during sea breeze events. Metropolitan locations such as Houston, Texas, have been intensely examined on a case-study basis (Banta et al. 2005; Banta et al. 2011), and will be the focus of the DISCOVER-AQ campaign in 2013. A climatological analysis of gulf breeze events would give an expanded view of historical air quality events in that region. Other possibilities include both urban and non-urban areas of New England, as more remote locations often experience the effects of transported pollutants downwind of major cities.
